# The bi-directional relationships between diversified leisure activity participation and cognitive function in older adults in China: separating between-person effects from within-person effects

**DOI:** 10.1186/s12877-024-04997-0

**Published:** 2024-05-13

**Authors:** Jingjing Wang, Shaojie Li, Yang Hu, Longbing Ren, Yuling Jiang, Mingzhi Yu, Zhouwei Liu, Yifei Wu, Yejin Zhao, Jie Zhang, Jing Li, Yao Yao

**Affiliations:** 1 School of Public Health, Peking University, Beijing, China; 2 Center for Health Development Studies, Peking University, Beijing, China; 3 Department of Geriatric Medicine, Beijing Hospital, National Center of Gerontology, Institute of Geriatric Medicine, Chinese Academy of Medical Sciences, Beijing, China

**Keywords:** Cognitive function, Leisure activity, Adjusted RI-CLPM, CLHLS

## Abstract

**Objective:**

To examine the bi-directorial association between diversified leisure activity participation and cognitive function over a 7-year period.

**Methods:**

Data analyzed was from the Chinese Longitudinal Healthy Longevity Survey (CLHLS), a large-scale longitudinal national study. The baseline survey was conducted in 2011 with follow-up every three years. We traced a total of 2718 participants over a period of 7 years. We used adjusted random intercept cross-lagged panel models (RI-CLPMs) to examine the bi-directorial associations between diversified leisure activity participation and cognitive function.

**Results:**

We observed bi-directorial associations between diversity of leisure activity and cognitive function across waves at the between-person and within-person levels. The adjusted random intercept cross-lagged panel models fitted the data appropriately, and the 3-year cross-lagged effects of prior diversified leisure activity participation on cognitive function (β = 0.058, *p* < 0.01) and cognitive function on subsequent diversified leisure activity participation (β = 0.047, *p* < 0.05) were significant. The results remained after adjusting the model for baseline sex, age, educational level, marital status and current residence, the number of chronic diseases, ADL, depressive symptoms, sleep quality, smoking, and drinking.

**Conclusion:**

This study suggests that a reciprocal causality relationship between diversified leisure activity participation and cognitive function, indicating a “positive circle” that further promotes cognition over time.

**Supplementary Information:**

The online version contains supplementary material available at 10.1186/s12877-024-04997-0.

## Introduction

As the world’s largest older population, China needs to achieve healthy aging to successfully deal with the economic and social challenges of rapid demographic aging [1]. However, dementia and mild cognitive impairment exhibit a high prevalence in China, with rates of 6.0% and 15.5% among the older population, respectively [2]. . It is necessary to develop strategies to lower the incidence of dementia and cognitive impairment among older adults. Based on cognitive reserve models, individuals can compensate for the progression of Alzheimer’s disease (AD) path by engaging in leisure activities that build skills or cognitive networks that are more efficient [3]. It is believed that experiencing and learning from diverse activities in everyday life will lead to an increase in cognitive reserve capacity and resilience, which will lead to better performance in cognitively challenging situations [4]. For that reason, diversified leisure activity may play a promising role in protecting older adults’ cognitive functions. In previous studies, participating in multiple leisure activities has been related to cognitive functioning [5, 6]. It is important for cognitive health in adulthood to have a variety of activities [4]. Engagement in a diverse array of leisure activities may confer a stronger protective effect against dementia in comparison to the frequent repetition of specific pursuits [7] .

In contrast, there has been limited research on the role of cognitive function in predicting the diversity of leisure activities. When compared to healthy individuals, those diagnosed with Alzheimer’s disease (AD) tend to engage less frequently in intellectual, passive, and physical activities during midlife [8]. A study conducted on a Japanese community-living sample of relatively healthy, cognitively unimpaired elderly individuals revealed a significant decline in leisure activity scores among the elderly as compared to the younger age group. Notably, with regard to cognitive domains, the scores for visuospatial and language abilities were strongly correlated with nonphysical hobby engagement [9]. In a recent investigation, the correlation between engaging in various recreational activities and cognitive function was explored, but the study did not distinguish between the within-person level and the between-person level [10]. Consequently, there is a pressing need for further investigation to bridge this knowledge gap.

The potential mechanism underlying the reciprocal relationship between participation in leisure activities and cognitive function have been proposed to mainly involve the cognitive reserve [11]. Cognitive reserve is a concept that is typically assessed through proxy variables that reflect a person’s lifetime experiences. Lifelong experiences are enriched by leisure activities. Increased participation in cognitively, socially, and physically engaging leisure activities has been linked to a decreased likelihood of mild cognitive impairment (MCI) and dementia, due to enhancements in cognitive reserve [12–14]. Conversely, based on the theory of planned behavior, health-related behaviors are influenced by cognitive reserve, all of which require cognitive functioning. Consequently, individuals with higher levels of cognitive function are more likely to engage in a variety of leisure activities [15]. Moreover, other potential mechanisms that may explain the relationship between engagement in leisure activities and cognitive function include the moderating influences of self-rated literacy level [10] and life course socioeconomic status [16].

The majority of empirical research demonstrating the association between participation in leisure activities and cognitive function is derived from cross-sectional studies, uni-directional longitudinal effect and between-person analyses (relative to others), thus limiting the ability to address questions regarding longitudinal within-person differences [10, 17–20]. It is not sufficient to provide a comprehensive understanding of the relationship between engagement in leisure activities and cognitive function. The reciprocal association between engagement in leisure activities and cognitive function may be observed at the individual level adopting a random intercept cross-lagged model. This relationship could be elucidated by posing the inquiry: Does an individual’s heightened participation in leisure activities (than they did in usual) correspond with a subsequent enhancement in cognitive function (a within-person difference)?

To address the aforementioned knowledge gaps we undertook a longitudinal analysis based on a nationwide prospective cohort, the Chinese Longitudinal Healthy Longevity Survey (CLHLS). Our primary aim was to explore the bidirectional relationship between the diversity of leisure activities and cognitive ability. Furthermore, we employed a Random Intercept Cross-Lagged Panel Model analysis to infer the causality in the above relationships.

## Methods

### Data source

The Chinese Longitudinal Healthy Longevity Survey (CLHLS) is a longitudinal cohort study representing a diverse sample. This study encompassed approximately 85% of the total population in 22 out of the 31 provinces by selecting half of all counties and cities. The dataset utilized in this study derives from the last three survey waves conducted under the CLHLS, each occurring at three-year intervals from 2011 to 2018.

It is important to note that the CLHLS was conducted with the approval of the Ethics Committee of Peking University (IRB00001052–13,074). In accordance with the study’s inclusion criteria, participants across the three waves were excluded if they did not meet the following conditions: (a) being aged 60 or older, and (b) having complete data for the key explanatory and outcome variables, specifically diversified leisure activity participation and cognitive function. The final analytical sample consisted of data from 2,718 participants who met these criteria across all three waves (2011, 2014, 2018) (The comparison between groups of final and original data. Please see the supplementary materials Table A1).

### Measurements

#### Cognitive function

Cognitive function was measured using the Chinese version of the modified Mini-Mental State Examination (CMMSE), which is widely accepted by researchers [21]. As a result of CLHLS being modified according to the actual situation of the elderly in China, it makes it easier for them to understand and answer the questions. There are 24 items in the CMMSE. There are 30 points available on the CMMSE; a higher score indicates better cognitive functioning. It has been verified by several previous studies that the Chinese MMSE is valid and reliable [22, 23]. In our study, the Chinese MMSE also has acceptable reliability and validity. For example, the Cronbach’s α of reliability for the Chinese MMSE in T1,T2 and T3 is 0.728,0.767 and 0.880. Moreover, the confirmatory factor analysis (CFA) shows that the Chinese MMSE in T1,T2 and T3 has good structural validity with acceptable fit across indices (**T1**:CFI > 0.95, TLI > 0.95, RMSEA < 0.05, SRMR < 0.05; **T2**:CFI > 0.95, TLI > 0.90, RMSEA < 0.08, SRMR < 0.05; **T3**:CFI > 0.95, TLI > 0.95, RMSEA < 0.08, SRMR < 0.05).

#### Diversified leisure activity participation

The definition of leisure activities is those in which individuals participate for their own pleasure and which are independent of their work and daily lives [24]. Diversified leisure activity participation was indicated by the number of activity participation in the following five aspects: garden work, read newspapers/books, play cards/mah-jongg, watch TV or listen to radio, social activities (to ensure that the 2011,2014,2018 three-wave data has complete information about these activities, we choose the above five activities). The total score ranged from 0 to 5. 0 (absent) and 1 (present: sometimes or more) for each activity, and sum these up ranging from 0 (none out of 5 activities) to 5 (all 5 activities). Higher scores indicated more greater diversity of leisure activity.

#### Covariates

In our analysis, we controlled for a number of covariates in 2011 baseline time, including demographic, health status and health behavior [25]. Demographic characteristics encompassed several variables, including sex (categorized as male or female), age group (ranging from 60 to 70, 71–80, 81–90, to 90+), educational attainment (grouped as 0 years, 1–6 years, or above 6 years), marital status (classified as married, divorced/widowed, or never married), and current place of residence (categorized as city, town, or rural).

Health status was assessed through the following indicators: the number of chronic diseases, activities of daily living (ADL) functioning, which includes a composite score reflecting independence in activities such as bathing, dressing, toileting, indoor transferring, continence, and eating, as well as scores indicating the presence of depressive symptoms. Health behavior was evaluated using the following measures: sleep quality scores, smoking status (dichotomized as either yes or no), and drinking habits (categorized as either yes or no).

### Statistic analysis

First, we conducted a descriptive analysis of the study population in order to describe its general characteristics. Secondly, we used zero-order correlations analysis among diversified leisure activity participation and cognitive function within and across waves. Thirdly, a traditional CLPM was compared to two random intercepts cross-lagged panel models (RI-CLPM and adjusted RI-CLPM). Orth et al. (2021) [26] propose the use of CLPM for analyzing between-person effects and the use of RI-CLPM for analyzing within-person effects, separate between-person effects from within-person effects. Mund and Nestler (2019) [27] have suggested the RI-CLPM to specifically account for pure within-person autoregressive and cross-lagged effects. As a result, the random intercepts capture the long-run, trait-like stability of the system. All models assessed the relationships between diversified leisure activity participation and cognitive function from 2011 to 2018 (T1 to T3: three-year lag time between 3 measurement points). Three models were built. The first model (Model 1) was a CLPM of diversified leisure activity participation and cognitive function used for examining between-person effects; the second model (Model 2) was a standardized constrained RI-CLPM of diversified leisure activity participation and cognitive function used for examining within-person effects and between-person effect; the third model (Model 3) was a standardized constrained RI-CLPM of diversified leisure activity participation and cognitive function that adjusted for baseline covariates used for examining within-person effects and between-person effect. The goodness of fit for both CLPM, RI-CLPM and adjusted RI-CLPM were comprehensively evaluated by using comparative fit index (CFI), Tucker-Lewis Index (TLI), and Root Mean Square Error of Approximation (RMSEA), (Standardized) Root Mean Square Residual (SRMR), and Akaike information criterion (AIC) and Bayesian information criteria (BIC). CFI, TLI values > 0.90 and RMSEA, SRMR < 0.08 suggest acceptable fit [28]. When comparing three models (CLPM VS RI-CLPM VS adjusted RI-CLPM), a lower AIC and BIC value indicates a superior model. A full-information maximum-likelihood (FIML) estimate was used for missing data of covariates. A sensitivity analysis was conducted to assess the robustness of the reciprocal relationship between engaging in leisure activities and cognitive function. For example, we excluded participants with MMSE scores < 21, as those with severe cognitive impairment may be more likely to recall incidents relating to engaging in leisure activities. All analyses were implemented in SPSS 27.0 and Mplus 8.3 and *P* < 0.05 indicates statistically significant.

## Results

### Descriptive statistics

Sample characteristics are shown in Table 1. The mean scores of this samples from 2011 to 2018 were 26.06, 25.27 and 22.66 for cognitive function, 1.78, 1.77 and 1.36 for diversified leisure activity participation .

**Table 1:** The characteristics of sample (*N* = 2,718)

Variables	Category	*N*	Mean ± SD /Percentage(%)
Age		2718	78.14 ± 8.87
Gender	Male	1314	48.3
	Female	1404	51.7
Educational level	0 year	1323	48.7
	1–6 Years	995	36.6
	above 6 years	392	14.4
	Missing	8	0.002
Marital status	married	1530	56.3
	divorced/widowed	1148	42.2
	never married	33	1.2
	Missing	7	0.3
Current residence	city	366	13.5
	town	798	29.4
	rural	1554	57.2
Smoking	Yes	563	20.7
	No	2141	78.8
	Missing	14	0.5
Drinking	Yes	563	20.7
	No	2127	78.3
	Missing	28	1.0
The number of chronic diseases		2718	1.21 ± 1.47
Activities of daily living		2650	97.5
	Missing	68	2.5
Depressive symptoms		2560	94.2
	Missing	158	5.8
Sleep quality		2717	100.0
	Missing	1	0.0
Diversified leisure activity participation T1		2718	1.78 ± 1.20
Diversified leisure activity participation T2		2718	1.77 ± 1.25
Diversified leisure activity participation T3		2718	1.36 ± 1.14
Cognitive function T1		2718	26.06 ± 5.04
Cognitive function T2		2718	25.27 ± 5.82
Cognitive function T3		2718	22.66 ± 8.55

### Correlations analysis

Table 2 illustrate the pairwise correlation of engagement leisure activity/cognitive function at the T1/ T2 /T3 time point, and the correlation of engagement leisure activity with cognitive function at the T1/ T2 /T3 time point. On any given occasion, diversified leisure activity participation and cognitive function were positively and significantly correlated (*p* < 0.001). The score of diversified leisure activity participation was positively correlated with later cognitive function (*p* < 0.001) and cognitive function was positively correlated with the later diversified leisure activity participation (*p* < 0.001).

**Table 2:** Correlations between diversity of leisure activity and cognitive function within and across waves

Variables	1	2	3	4	5	6
Diversified leisure activity participation T1	1	0.528***	0.448***	0.309***	0.248***	0.279***
Diversified leisure activity participation T2		1	0.497***	0.287***	0.324***	0.310***
Diversified leisure activity participation T3			1	0.277***	0.283***	0.436***
Cognitive function T1				1	0.418***	0.394***
Cognitive function T2					1	0.451***
Cognitive function T3						1

^*^P < 0.05;^**^P < 0.01;^***^P < 0.001

### CLPM VS RI-CLPM VS adjusted RI-CLPM

Table 3 provides a summary of the fit indices for the three models. Model 1 was a CLPM of diversified leisure activity participation and cognitive function. The model displayed poor fit across indices (CFI > 0.9, TLI < 0.9, RMSEA > 0.08, SRMR < 0.08), suggesting that the specified model is not suitable for the given data. Model 2 was a standardized constrained RI-CLPM of diversified leisure activity participation and cognitive function. The model also displayed unsatisfactory fit across indices (CFI > 0.9, TLI < 0.9, RMSEA > 0.08, SRMR < 0.08), suggesting that the specified model is not suitable for the given data. Model 3 was adjusted standardized constrained RI-CLPM of diversified leisure activity participation and cognitive function with baseline covariates (a series of demographic, health status and health behavior variables). The model exhibited acceptable fit across indices (CFI > 0.9, TLI > 0.9, RMSEA < 0.08, SRMR < 0.08,a lower AIC and BIC value), indicating that the specified model does fit the data.

**Table 3:** Model fit indices

	χ2	df	CFI	TLI	RMSEA	SRMR	AIC	BIC
Model 1	266.730	4	0.915	0.704	0.155	0.054	75517.046	75652.922
Model 2	140.942	5	0.960	0.880	0.100	0.054	75354.602	75484.570
Model 3	294.187	49	0.941	0.902	0.047	0.052	61151.594	61403.760

Model 1 = cross-lagged panel model (CLPM)

Model 2 = standardized constrained random intercepts cross-lagged panel model (RI-CLPM)

Model 3 = standardized constrained RI-CLPM adjusting for baseline age, sex, educational level, marital status, current residence, the number of chronic diseases, activities of daily living, depressive symptoms, sleep quality scores, smoking, and drinking

### The reciprocal relationship between diversified leisure activity participation and cognitive function

**Fig. 1:**
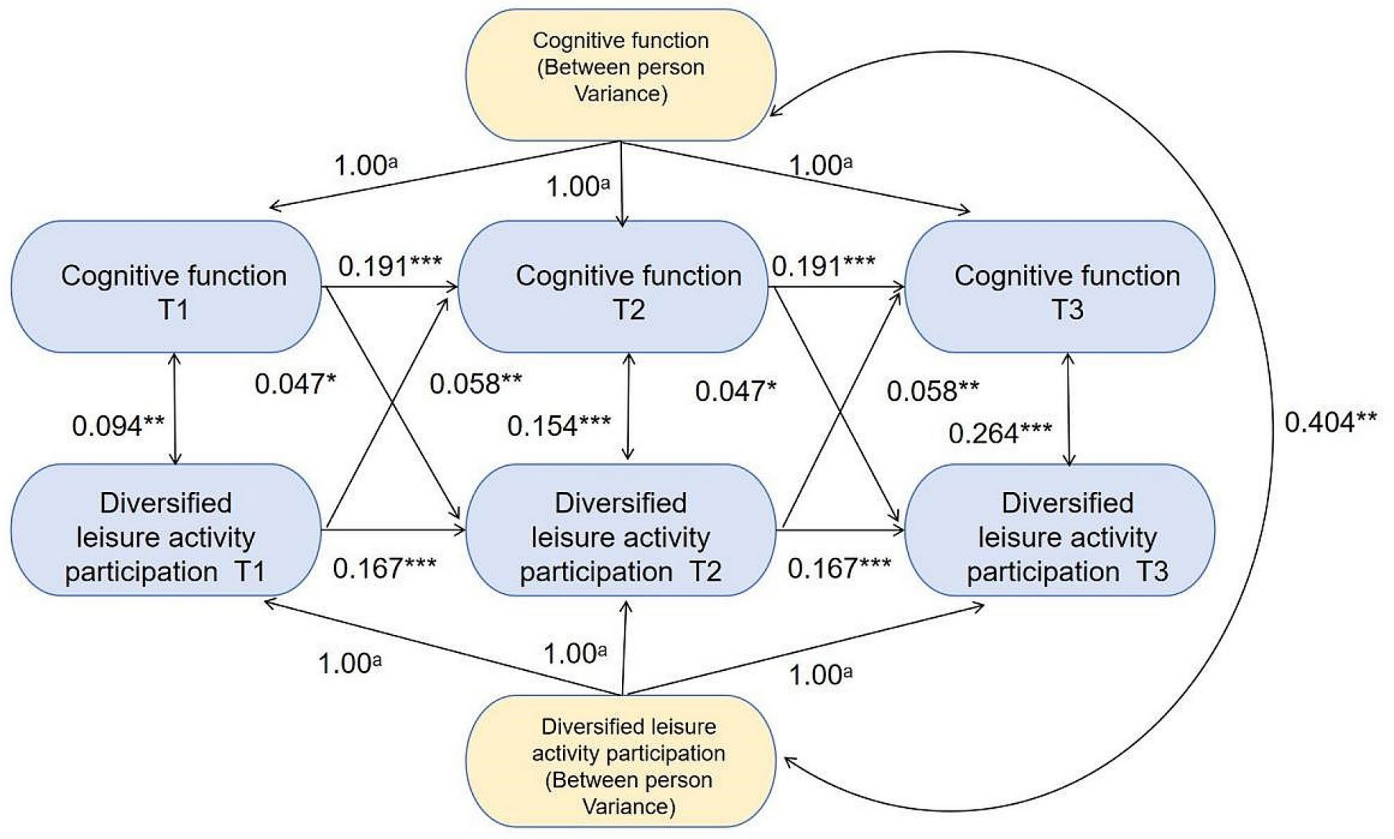
Random-Intercepts, Cross-Lagged Panel Model Illustrating Within-Person Association Between Type diversity of leisure activity and Cognitive function, Controlling for baseline age, sex, educational level, marital status, current residence, the number of chronic diseases, activities of daily living, depressive symptoms, sleep quality scores, smoking and drinking. a Pathways constrained to 1.00 to isolate between-person factor; *p < 0.05, **p < 0.01, ***p < 0.001

After controlling for covariates (demographic characteristics (sex, age, educational level, marital status and current residence), health status (the number of chronic diseases, ADL, depressive symptoms), health behavior (sleep quality, smoking, and drinking)), Model 3 (Fig. 1) fitted the data appropriately, and the 3-year cross-lagged effects of prior diversified leisure activity participation on cognitive function (β = 0.058, *p* < 0.01) and cognitive function on subsequent diversified leisure activity participation (β = 0.047, *p* < 0.05) were significant (within-person level) (Table 4). Higher prior diversified leisure activity participation predicted higher subsequent cognitive function, and higher prior cognitive function predicted higher subsequent diversified leisure activity participation. Moreover, the between-person association between leisure activities and cognitive function was strong and positive, indicating that those with higher levels of engagement in leisure activities across multiple measurement waves also reported higher levels of cognitive function compared to those with lower levels of engagement (β = 0.404, *p* < 0.01).

**Table 4:** Standardized coefficients of leisure activities and cognitive function

Parameter	Model1		Model2		Model3	
	β	SE	β	SE	β	SE
Between-person	/	/	0.580***	0.037	0.404**	0.126
Stability paths						
LA1→LA2	0.485***	0.017	0.225***	0.031	0.167***	0.030
LA2→LA3	0.453***	0.017	0.225***	0.031	0.167***	0.030
CF1→CF2	0.377***	0.027	0.160***	0.043	0.191***	0.040
CF2→CF3	0.392***	0.023	0.160***	0.043	0.191***	0.040
Cross-lagged effects						
LA1→CF2	0.131***	0.018	0.116***	0.024	0.058**	0.021
LA2→CF3	0.183***	0.016	0.116***	0.024	0.058**	0.021
CF1→LA2	0.137***	0.016	0.082**	0.025	0.047*	0.024
CF2→LA3	0.137***	0.015	0.082**	0.025	0.047*	0.024
Synchronous Correlations						
LA1 WITH CF1	0.309***	0.016	0.127**	0.037	0.094**	0.032
LA2 WITH CF2	0.194***	0.018	0.196***	0.025	0.154***	0.025
LA3 WITH CF3	0.311***	0.016	0.308***	0.020	0.264***	0.021

LA: leisure activity; CF: cognitive function

Model 1 = cross-lagged panel model (CLPM)

Model 2 = standardized constrained random intercepts cross-lagged panel model (RI-CLPM)

Model 3 = standardized constrained RI-CLPM adjusting for baseline age, sex, educational level, marital status, current residence, the number of chronic diseases, activities of daily living, depressive symptoms, sleep quality scores, smoking, and drinking

*p < 0.05, **p < 0.01, ***p < 0.001

### Sensitivity analyse

The sensitivity analysis indicates that the reciprocal associations between engaging in leisure activities and cognitive function remained significant after excluding participants with severe cognitive impairment and MMSE scores < 21. The 3-year cross-lagged effects of prior diversified leisure activity participation on cognitive function (β = 0.075, *p* < 0.001) and cognitive function on subsequent diversified leisure activity participation (β = 0.048, *p* < 0.05) were significant.

## Discussion

In our knowledge, this is the first large-scale cohort study that examines the reciprocal prospective relationship between diversified leisure activity participation and cognitive function in Chinese elderly, separate between-person effects from within-person effects. Moreover, our findings demonstrate that cognitive improvement may enhance diversified leisure activity participation by strengthening the assertion that diversified leisure activity participation has a positive impact on cognitive function, creating a “positive circle” that further promotes cognition over time. In other words, It was found that changes in diversified leisure activity participation predicted changes in typical cognitive function and an individual’s typical cognitive ability predicted their participation in diversified leisure activities. Consistent findings were obtained after adjusting for baseline age, sex, educational level, marital status, current residence, the number of chronic diseases, activities of daily living, depressive symptoms, sleep quality scores, smoking and drinking.

There was a slight difference in findings when using the traditional CLPM versus the RI-CLPM versus adjusted RI-CLPM, whereby the CLPM and RI-CLPM found larger associations between diversified leisure activity participation and cognitive function across all three waves but have poor model fit indices. The adjusted RI-CLPM suggests a slight small but significant association between diversified leisure activity participation and cognitive function across all three waves and shows an acceptable model fit index. Therefore, the adjusted RI-CLPM at the within-person level and between-person level was chosen as the primary analysis for our study.

The present study identified the significant effect of engaging in leisure activities on cognitive function overtime at a within-person level and between-person level in a longitudinal population-based cohort, separate between-person effects from within-person effects. The findings of the adjusted RI-CLPMs suggest that individuals may experience a subsequent increase in cognitive function when engaging in higher levels of leisure activities than usual, after adjusting for a series of covariates [26]. No research has been conducted to investigate the impact of alterations in leisure activities on cognitive function at a within-person level. Prior research on the relationship between leisure activities and cognitive function has indicated that alterations in leisure activities can impact cognitive function at the between-person level [10]. Additional research is required to confirm the results of our study on the relationship between leisure activities and cognitive function at a within-level level.

In most previous studies, a unidirectional effect of diversified leisure activity participation on cognitive function has been found [5, 29]. Through an examination of a nationally representative longitudinal dataset, we demonstrate a positive bidirectional causality between diversified leisure activity participation and cognitive function. A bidirectional relationship between diversified leisure activity participation and cognitive function over time was still significant, even after accounting for demographic characteristics (sex, age, educational level, marital status and current residence), health status (the number of chronic diseases, ADL, depressive symptoms), health behavior (sleep quality, smoking, drinking). Accordingly, these variables do not explain the reciprocal relationship between diversified leisure activity participation and cognitive function. By excluding these covariates, we rule out some third-variable effects of confounding variable that distorts the casual impact of diversified leisure activity participation on cognitive function [30] .

Engaging in a diverse range of activities, irrespective of their frequency, was found to be positively correlated with the preservation of cognitive function in older age [31]. According to Carlson et al. (2012), engaging in a diverse range of activities is a more reliable predictor of clinically significant reductions in the risk of developing impairment in immediate and delayed verbal memory, as well as global cognition, which are outcomes indicative of dementia risk. Specifically, for each additional activity endorsed, regardless of frequency, participants experienced an 8–11% decrease in their risk of developing memory and global cognitive impairment. This study posits that while increasing the frequency of cognitively challenging activities per month is important, it may not be as critical as diversifying the total number and variety of activities in order to support cognitive health. Exposure to a range of lifestyle activities with varying levels of cognitive stimulation can be viewed as a proxy for environmental enrichment and complexity in older age. The complexity of one’s environment plays a role in promoting brain plasticity and cognitive reserve [13, 32] .

According to the cognitive reserve hypothesis, some people are able to cope with certain neuropathology such as AD through their intelligence or life experiences like leisure activities [33]. There also was a correlation between greater activity diversity and greater hippocampal volume on average across the left and right hemispheres [34]. Research on animal models of enriched environments has shown that cognitive, sensory and physical activities are enhanced, as well as the neurobiological bases of brain function and memory [35]. These may well explain the effect of activity type diversity on cognition. However, the effect of cognition on activity type diversity is rarely considered. A study using CLPM at the between-person level illustrates the reciprocal relationship between participation in leisure activities and cognitive ability [10]. In our study, the beneficial effect of cognitive improvement on the diversified leisure activity participation has also been found. In previous a cross-sectional study, there were significantly lower scores for type of daily activities and cognitive function in the frail older adult group [36]. Moreover, according to the theory of planned behavior, health-related behavior can be predicted by intention, perceived behavior control, and attitude, all of which need cognitive function to play a role [37]. Therefore, people with high cognitive function will take part in more kinds of leisure activities. Further research would be required to study this issue.

One notable finding from our model deserves attention. In contrast to self-prediction of cognition or leisure activity over time, cross-prediction between these two variables were apparently weaker. Firstly, autoregressive effects of cognition or leisure activity refer to the degree of consistency in constructs across successive time points. Smaller autoregressive coefficients, which are closer to zero, suggest greater variance in the construct, indicating diminished stability or influence from the preceding time point. Conversely, larger autoregressive coefficients imply minimal variance over time, signifying heightened stability or influence from the preceding time point [38, 39]. Secondly, cross-lagged effects reflect the effect of a construct on another measured at a later time. Individual differences in the constructs determine the size of these effects [38]. Moreover, according to statistical theory, the magnitude of cross-lagged effects is contingent upon the temporal interval between assessments, with effect sizes potentially fluctuating in accordance with the duration of this interval [40].

Moreover, in terms of applications, within-person level correlations may aid in pinpointing actionable areas for intervention, whereas between-person level correlations are instrumental in identifying individuals who may benefit from intervention [41, 42]. In our study, We employed the adjusted RI-CLPM to effectively model the data and found a significant within-person and between-person effect. The within-person findings of our research offer understanding of the reciprocal relationship between leisure activities and cognitive function within individual elderly. Elderly individuals who engage in fewer leisure activities than their did in usual may be susceptible to a decline in cognitive function. This highlights the importance of closely monitoring individual daily leisure activities. In addition, the between-person results indicate elderly individuals with lower levels of engagement in leisure activities compared to their peers are more likely to exhibit poor cognitive function. It may prompt that engaging in peer comparisons of leisure activities may have a significant impact on cognitive function.

This study presents several strengths, notably the utilization of three-wave longitudinal data, enabling the examination of the bidirectional relationship between the diversity of leisure activities and cognitive function. This approach elucidates the creation of a “positive circle” where improvements in cognition and increased participation in leisure activities mutually reinforce one another over time. However, it is important to acknowledge certain limitations in this study. Although it is inevitable to have a small percentage of missing data on covariates in large longitudinal studies, particularly those based on a national population sample, careful consideration should be given when interpreting the findings. While we have taken into account a comprehensive set of significant covariates, some unmeasured factors, such as personality traits [43, 44], were not included in our analysis. Future research endeavors could explore the influence of personality traits on the connection between activity diversity and cognitive functioning.

## Conclusion

Utilizing an adjusted random-intercepts cross-lagged panel model on analyzing a nationwide data of older adults in China, our study revealed a positive and reciprocal causal association between diversified leisure activity participation and cognitive ability at the within-person level and between-person level, separate between-person.

effects from within-person effects. This relationship remained consistent even after accounting for the influence of demographic and health-related characteristics, indicating a “positive circle” that continues to enhance cognitive function over time. These findings suggest that future interventions designed to improve cognitive function should encompass a diverse range of activities, and conversely, promoting cognitive function may, in turn, foster engagement in a wider array of leisure activities.

### Electronic supplementary material

Below is the link to the electronic supplementary material.

Supplementary Material 1

## Data Availability

The datasets used and/or analysed during the current study available from the corresponding author on reasonable request.
