# Supplementary material for: The bi-directional relationships between diversified leisure activity participation and cognitive function in older adults in China: separating between-person effects from within-person effects
Source: BMC Geriatr. 2024 May 13;24:426. doi: 10.1186/s12877-024-04997-0 (PMC11092250; doi:10.1186/s12877-024-04997-0)
Supplement: Supplementary file 1 — Supplementary Material 1 [file 12877_2024_4997_MOESM1_ESM.docx]

Supplementary materials

**TABLE A1. Group comparison between final sample and original sample.**

| Variables | Category | Final sample  （N） | Original sample  (N) | P for T  /P for χ² |
| --- | --- | --- | --- | --- |
| Age |  | 2718 | 9765 | <0.001 |
| Gender | Male | 1314 | 4398 | 0.002 |
|  | Female | 1404 | 5367 |  |
| Educational level | 0 year | 1323 | 5652 | <0.001 |
|  | 1-6 Years | 995 | 2967 |  |
|  | above 6 years | 392 | 1105 |  |
|  | Missing | 8 | 41 |  |
| Marital status | married | 1530 | 3698 | <0.001 |
|  | divorced/widowed | 1148 | 5868 |  |
|  | never married | 33 | 101 |  |
|  | Missing | 7 | 98 |  |
| Current residence | city | 366 | 1735 | <0.001 |
|  | town | 798 | 2885 |  |
|  | rural | 1554 | 5145 |  |
| Smoking | Yes | 563 | 1733 | <0.001 |
|  | No | 2141 | 7928 |  |
|  | Missing | 14 | 104 |  |
| Drinking | Yes | 563 | 1640 | <0.001 |
|  | No | 2127 | 7972 |  |
|  | Missing | 28 | 153 |  |
| The number of chronic diseases |  | 2718 | 9765 | <0.001 |
| Activities of daily living |  | 2650 | 9465 | <0.001 |
|  | Missing | 68 | 300 |  |
| Depressive symptoms |  | 2560 | 8141 | <0.001 |
|  | Missing | 158 | 1624 |  |
| Sleep quality |  | 2717 | 9736 | 0.389 |
|  | Missing | 1 | 29 |  |
| Diversified leisure activity participation T1 |  | 2718 | 9669 | <0.001 |
|  | Missing | 0 | 96 |  |
| Diversified leisure activity participation T2 |  | 2718 | 5897 | <0.001 |
|  | Missing | 0 | 3868 |  |
| Diversified leisure activity participation T3 |  | 2718 | 2777 | 0.945 |
|  | Missing | 0 | 6988 |  |
| Cognitive function T1 |  | 2718 | 9765 | <0.001 |
|  | Missing | 0 | 0 |  |
| Cognitive function T2 |  | 2718 | 6066 | <0.001 |
|  | Missing | 0 | 3699 |  |
| Cognitive function T3 |  | 2718 | 2884 | 0.18 |
|  | Missing | 0 | 6881 |  |
